# Autotaxin is a novel target of microRNA‐101‐3p

**DOI:** 10.1002/2211-5463.12608

**Published:** 2019-03-01

**Authors:** Yuqin Wang, Lin Lyu, Xiaotian Zhang, Junjie Zhang

**Affiliations:** ^1^ The Key Laboratory of Cell Proliferation and Regulation Biology Ministry of Education Institute of Cell Biology College of Life Sciences Beijing Normal University China

**Keywords:** autotaxin, cancer cell, invasion, migration, miR‐101‐3p, proliferation

## Abstract

Autotaxin (ATX), a vital enzyme that generates lysophosphatidic acid (LPA), affects many biological processes, including tumorigenesis, via the ATX–LPA axis. In this study, we demonstrate that microRNA‐101‐3p (miR‐101‐3p), a well‐known tumor suppressor, downregulates ATX expression at the posttranscriptional level. We found that miR‐101‐3p inhibits ATX regulation by directly targeting a conserved sequence in the ATX mRNA 3′UTR. Moreover, we observed an inverse correlation between ATX and miR‐101‐3p levels in various types of cancer cells. ATX is highly expressed in several human cancers. Here, we verified that ATX expression is significantly inhibited by miR‐101‐3p in U87 and HCT116 cells. ATX downregulation contributed to the suppression of migration, invasion, and proliferation mediated by miR‐101‐3p; furthermore, the tumor‐suppressing activity of miR‐101‐3p was partially reduced by the addition of LPA in U87 cells. Our data suggest that ATX is a novel target of miR‐101‐3p.

**Abbreviations**
ATX: autotaxin
CM: culture medium
LPA: lysophosphatidic acid
LPC: lysophosphatidylcholine
LysoPLD: lysophospholipase D
miR‐101‐3p: microRNA‐101‐3p

Autotaxin (ATX), also known as nucleotide pyrophosphatase/phosphodiesterase 2, is an extracellular enzyme with lysophospholipase D (LysoPLD) activity that catalyzes the hydrolysis of lysophosphatidylcholine (LPC) into lysophosphatidic acid (LPA) 1. ATX‐deficient mice are embryonically lethal due to impaired neurogenesis and vasculogenesis. ATX heterozygous mice develop normally, but their plasma LPA levels are reduced by half compared with wild‐type mice. 2. Thus, ATX is a vital enzyme that produces LPA in the blood. As a bioactive lysophospholipid, LPA participates in various cellular processes, including cell survival, proliferation, and migration, via binding to its specific G protein‐coupled receptors (LPAR1 to LPAR6) 3, 4. The majority of the biological functions of ATX are mediated by ATX–LPA signaling. Growing evidence indicates that the ATX–LPA axis is of great significance in many physiological and pathological processes, ranging from inflammatory diseases 5 and obesity 6 to tumorigenesis 7.

Autotaxin, as a tumor cell motility factor, was initially isolated from the conditioned medium of the human melanoma cell line A2058 8. ATX is frequently highly expressed in several human cancers, such as hepatocellular carcinoma, breast cancer, and neuroblastoma 9. Overexpression of ATX promotes tumorigenesis and metastasis in ras‐transformed NIH3T3 cells 10. ATX is primarily responsible for the motility of MDA‐MB‐435 cells 11, and the ectopic expression of ATX and LPA receptors increases mammary tumorigenesis and metastasis 7. As one of the top 40 most upregulated genes in metastatic cancer, ATX is considered a potential target in cancer therapy 12.

The regulation of ATX expression in cancer cells has been widely studied because of the biological significance of the ATX–LPA axis in tumorigenesis. ATX expression is increased by the transcription factor Sp3 in neuroblastoma cells 13. NFAT1 and STAT3 upregulate the expression of ATX by binding to the promoter of the *ATX* gene in breast cancer cells 11, and ATX expression is also promoted by TNF‐α in human hepatocellular carcinoma 14. In addition, many other cytokines, including VEGF, EGF, bFGF, and BMP‐2, have been reported to regulate ATX in cancer cells 15, 16, 17. Our previous study indicated that ATX expression is regulated by HDACs (HDAC3 and HDAC7) in various cancer cells 18. We have recently reported that ATX expression in cancer cells is regulated at the posttranscriptional level by the RNA‐binding proteins HuR and AUF1 19.

MicroRNAs (miRNAs) are short noncoding RNAs with ~ 22 nucleotides that usually downregulate the expression of target genes by cleaving mRNA and/or repressing mRNA translation 20. It has been reported in miRNA expression profiling studies that different cancers exhibit characteristic miRNA signatures 21. Increasing evidence indicates that miRNAs are involved in the regulation of tumorigenesis by functioning as either oncogenes or tumor suppressors 22. Regarding the significance of ATX in tumorigenesis and its potential as a therapeutic target for cancer, determining miRNA(s) that regulate ATX expression may contribute to the development of novel therapeutic approaches in cancer therapy. In this study, we demonstrate that ATX is a direct target of microRNA‐101‐3p (miR‐101‐3p), a well‐known tumor suppressor. Through targeting a conserved sequence in ATX mRNA 3′UTR, miR‐101‐3p downregulates ATX expression in cancer cells. The downregulation of ATX contributes to the tumor‐suppressing activity of miR‐101‐3p by suppressing cancer cell migration, invasion, and proliferation.

## Materials and methods

### Cell culture and transfection

HT29 and HCT116 cells were cultured in McCoy's 5A medium (CM10050; M&C Gene Technology, Beijing, China). MCF7, HeLa, HEK293, and U87 cells were maintained in Dulbecco's modified Eagle's medium (CM10013; M&C Gene Technology). All media were supplemented with 10% FBS (10099‐141; Thermo Fisher Scientific, Waltham, MA, USA), 100 U·mL^−1^ penicillin, and 100 μg·mL^−1^ streptomycin (15140122; Thermo Fisher Scientific). Cells were cultured in a humidified atmosphere containing 5% CO_2_ at 37 °C. Transfections of RNA oligoribonucleotides were performed using Lipofectamine RNAiMAX (13778‐150; Invitrogen, Carlsbad, CA, USA). RNA duplexes were used at a final concentration of 100 nm, and miRNA inhibitors were used at a final concentration of 200 nm in this study. Cotransfections of microRNA mimics and plasmids were performed using Lipofectamine 2000 (11668‐019; Invitrogen) according to the manufacturer's instructions. All siRNAs and microRNA mimics were synthesized by GenePharma (Shanghai, China). The sequences of RNA oligoribonucleotides were as follows: NC, 5′‐GGCUGCUGUGUAGAUCUCU‐3′; siDicer, 5′‐UGCUUGAAGCAGCUCUGGA‐3′; siPgrp, 5′‐UGUGCAGCACUACCACAUG‐3′; siATX, 5′‐GUGGACCAAUCUUCGACUA‐3′; microRNA inhibitor NC: 5′‐CAGUACUUUUGUGUAGUACAA‐3′; and miR‐101‐3p inhibitor: 5′‐UUCAGUUAUCACAGUACUGUA‐3′. The plasmid expressing pre‐miR‐101 was purchased from GenePharma, and the sequence corresponding to pre‐miR‐101 was ACTGTCCTTTTTCGGTTATCATGGTACCGATGCTGTATATCTGAAAGGTACAGTACTGTGATAACTGAAGAATGGTGGT.

### Reagents and antibodies

The 18:1 LPA was obtained from Avanti Polar Lipid Inc. (857230; Alabaster, AL, USA). The ATX antibody was generated in our laboratory as described previously 23. The antibodies used were specific for EZH2 (#5246; Cell Signaling Technology, Beverly, MA, USA) and β‐actin (sc‐47778; Santa Cruz Biotechnology, Santa Cruz, CA, USA).

### Plasmid construction

The pTRE‐d2EGFP‐ATX 3′UTR reporter plasmid was constructed with the full‐length ATX 3′UTR cloned downstream of the EGFP ORF in the pTRE‐d2EGFP vector (Clontech Laboratories, Palo Alto, CA, USA). The luciferase reporter plasmid was constructed by cloning the full‐length ATX 3′UTR immediately downstream of the *Renilla* luciferase ORF in the psiCHECK2 vector (Promega, Madison, WI, USA), termed pRLuc‐ATX‐3′UTR. A mutation was made in the predicated miR‐101‐3p binding site in the human ATX 3′UTR of pRLuc‐ATX‐3′UTR to create pRLuc‐ATX‐3′UTR‐mut.

### Luciferase assay

pRLuc‐ATX‐3′UTR and pRLuc‐ATX‐3′UTR‐mut were cotransfected separately with miR‐101‐3p or a control miRNA (miR‐NC) duplex into the indicated cells. Cell lysates were collected 48 h after transfection. *Renilla* and firefly luciferase activities were detected with a Dual‐Luciferase Reporter System Kit (E1910; Promega) following the manufacturer's instructions. The activity of *Renilla* luciferase in each sample was normalized to that of firefly luciferase.

### Western blot analyses

Cells were lysed in RIPA buffer for 20 min, and then, the supernatants were measured by bicinchoninic acid assays (#23225; Pierce Biotechnology, Rockford, IL, USA). Total proteins were loaded equally for each sample. For the preparation and detection of secreted ATX, cells were rinsed twice with phosphate‐buffered saline after transfection for 24 h and were then cultured with serum‐free conditional culture medium (CM) for another 24 h. CM was concentrated (30‐fold) using an Amicon Ultra centrifugation filter (UFC503096; Merck Millipore, Billerica, MA, USA) with a 30 K cutoff and equal loaded volume. SDS/PAGEs were performed and then analyzed with anti‐ATX, anti‐EZH2, or anti‐β‐actin antibody.

### RNA extraction and RT‐qPCR

Total RNA was extracted from cells with TRIzol reagent (T9424; Sigma‐Aldrich, St. Louis, MO, USA), and DNA was removed with RNase‐free DNase I (EN0521; Thermo Fisher Scientific). mRNA levels were evaluated by real‐time PCR with GAPDH as an internal control. The primers used for PCR amplification were as follows: for ATX, 5′‐GGATTGAAGCCAGCTCCTAAT‐3′ and 5′‐GCAACTGGTCAGATGGTCAGG‐3′; for GAPDH, 5′‐TTAGCACC CCTGTCCAAGG‐3′ and 5′‐CCTACTCCTTGGAGGCCATG‐3′; for Dicer, 5′‐TTAACCAGCTGTGGGGAGAGGGCTG‐3′ and 5′‐AGCCAGCGATGCAAAGATGGTGTTG‐3′; for Pgrp, 5′‐GCCCTGAGGTCCAACTATGT‐3′ and 5′‐AGCGGTAGTGTGGCCAATTC‐3′; for *Renilla* luciferase, 5′‐AAGAGCGAAGAGGGCGAGAA‐3′ and 5′‐TGCGGACAATCTGGACGAC‐3′; for firefly luciferase, 5′‐CGTGCCAGAGTCTTTCGACA‐3′ and 5′‐ACAGGCGGTGCGATGAG‐3′; and for EGFP, 5′‐TACAACTACAACAGCCACAACG‐3′ and 5′‐ATCCTGTCCTCCACCTCC‐3′. For the detection of miR‐101‐3p levels, total RNA was reverse‐transcribed using specific miRNA reverse primer and amplified with corresponding miRNA primers for qPCR that were provided by Ribo (RIBO Bio Inc., Guangzhou, China). The miR‐101‐3p levels were normalized to the levels of U6 to yield a $2^{-\Delta \Delta C_{t}}$ value.

### Migration and invasion assay

For Transwell migration assay, U87 cells was performed as described previously 19. Briefly, cells were starved for 8 h after RNA oligoribonucleotide transfection for 40 h. Then, U87 cells (2 × 10^4^) suspended in serum‐free DMEM were added to the upper chamber of the 24‐well Transwell chambers with 8‐μm‐pore‐size polycarbonate membrane (3422; Corning Costar, Cambridge, MA, USA). The 10% FBS DMEM was added to the lower chamber. Then, 18:1 LPA (2 μm) was added to the medium in the upper and lower chambers as indicated. Cells that migrated to the lower side of the upper chamber were fixed with 4% PFA (paraformaldehyde) and stained with hematoxylin after 24 h of cultivation. Then, the cells were imaged per field (200×) by microscopy and enumerated in 3 randomly chosen fields. The Transwell invasion assay was performed as same as above, except that the polycarbonate membrane was coated by Matrigel (#356234; BD Biosciences, Bedford, MA, USA) before adding U87 cells to upper chamber.

### Wound‐healing assay

For the wound‐healing assay, U87 cells that were transfected for 24 h were seeded in a 96‐well plate to ~ 90% confluence. Wound Maker generated wounds in the middle of each well after cells were starved for 12 h. Then, the cells were cultured in serum‐free medium in the absence or presence of 18:1 LPA (2 μm) as indicated. The percentage of relative wound density was analyzed with the incucyte zoom software (Essen Bioscience, Irvine, CA, USA).

### Cell proliferation assay

For the proliferation assay, U87 cells that were transfected for 24 h were seeded in a 96‐well plate to ~ 40% confluence. The cells were cultured with or without 18:1 LPA (10 μm) after starving for 12 h. Cell proliferation was monitored continuously with the incucyte zoom apparatus (Essen Bioscience).

## Results

### Screening for the miRNAs regulating ATX expression

**Figure 1:**
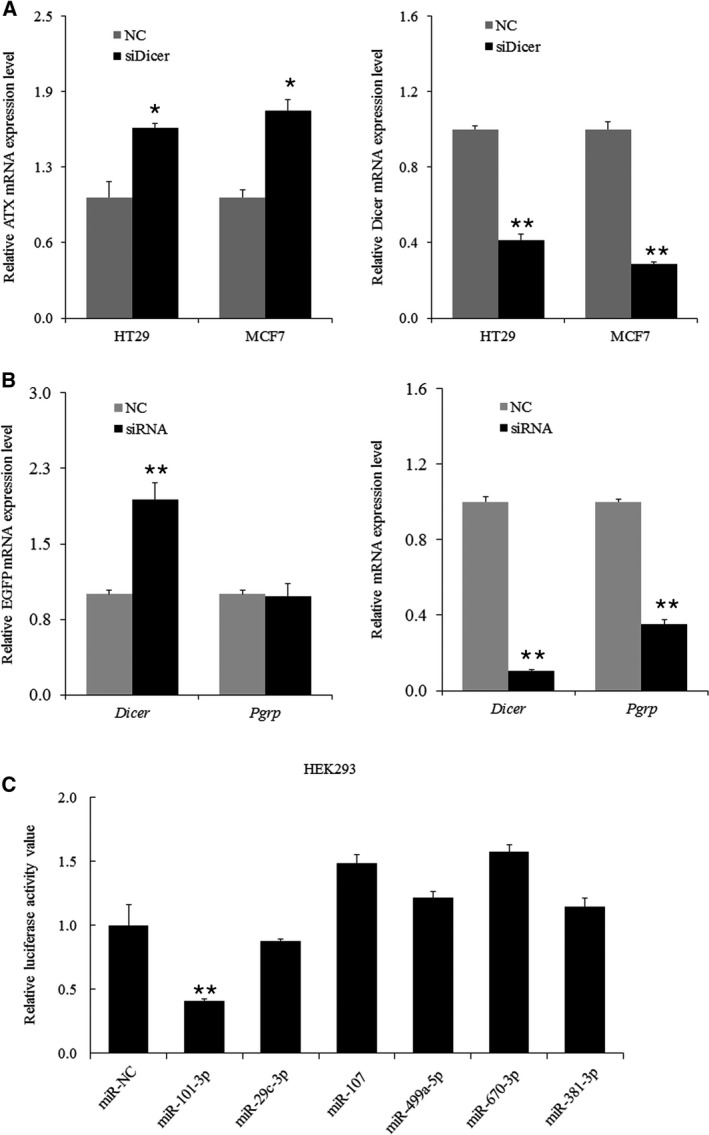
Identification of candidate miRNAs targeting ATX. (A) HT29 and MCF7 cells were transfected with siDicer duplexes or negative control siRNA (NC). RNA was isolated from the cells 48 h after transfection and then subjected to RT‐qPCR to assess ATX mRNA levels (left) and Dicer mRNA levels (right). (B) HeLa cells were cotransfected with pTRE‐d2EGFP‐ATX 3′UTR reporter plasmid and *Dice*r siRNA, an unrelated gene *Pgrp* siRNA, or a nonspecific siRNA (NC) as indicated. At 48 h after transfection, RT‐qPCR was performed to assess the levels of reporter EGFP mRNA (left), *Dicer* mRNA, and *Pgrp* mRNA (right). (C) HEK293 cells were cotransfected with the pRLuc‐ATX‐3′UTR plasmid and the indicated miRNA mimic or negative control (miR‐NC). A luciferase activity assay was conducted 48 h after transfection. Data are representative of three independent experiments. The error bars represent ± SEM. Statistical significance was determined using Student's *t*‐test. *P <* *0.05, **P <* *0.01.

To investigate whether the expression of the *ATX* gene is regulated by miRNAs, we used siRNA to silence *Dicer*, an RNase III family member that plays a key role in processing small RNAs in miRNA systems 24, 25, generating global suppression of miRNA maturation. As shown in Fig. 1A, downregulation of Dicer significantly increased ATX mRNA levels in HT29 and MCF7 cells, suggesting that miRNA(s) might be involved in regulating ATX expression. When the reporter plasmid pTRE‐d2EGFP‐ATX 3′UTR was cotransfected together with the siRNA specific to *Dicer* or *Pgrp* (peptidoglycan recognition protein LC, a gene unrelated to the miRNA system) into HeLa cells 26, the expression level of the reporter EGFP containing *ATX* 3′UTR was markedly increased when cells were treated with RNAi of *Dicer* but not *Pgrp* (Fig. 1B), suggesting that the miRNA system regulates *ATX* expression via its 3′UTR. To determine the potential miRNAs that directly target the human *ATX* 3′UTR, a search for putative binding sites of miRNAs in the 3′UTR of the human *ATX* gene was performed with targetscan, an online computational algorithm (http://www.targetscan.org). We listed the top 6 miRNAs that potentially targeted the *ATX* 3′UTR predicted by targetscan according to the conservation of species and context scores (Table 1). These candidate miRNAs were further tested by luciferase reporter assays. HEK293 cells were cotransfected with each candidate miRNA together with a reporter plasmid containing *ATX* 3′UTR (pRLuc‐ATX‐3′UTR). A significant reduction in luciferase activity was detected when the cells were treated with microRNA‐101‐3p (miR‐101‐3p) (Fig. 1C). These data suggest that ATX expression can be downregulated by miRNA and that miR‐101‐3p is a potential miRNA that targets the *ATX* 3′UTR.

**Table 1:** The top six putative miRNAs targeting human ATX

miRNA	Total putative binding sites	8‐mer binding site	7‐mer‐m8 binding site	7‐mer‐1A binding site	Total context score
miR‐101‐3p	1	1			−0.33
miR‐29‐3p	1		1		−0.32
miR‐103‐3p/107	1			1	−0.22
miR‐499a‐5p	1		1		−0.19
miR‐670‐3p	1	1			−0.43
miR‐381‐3p	1			1	−0.14

### miR‐101‐3p targets a conserved sequence in ATX mRNA 3′UTR

**Figure 2:**
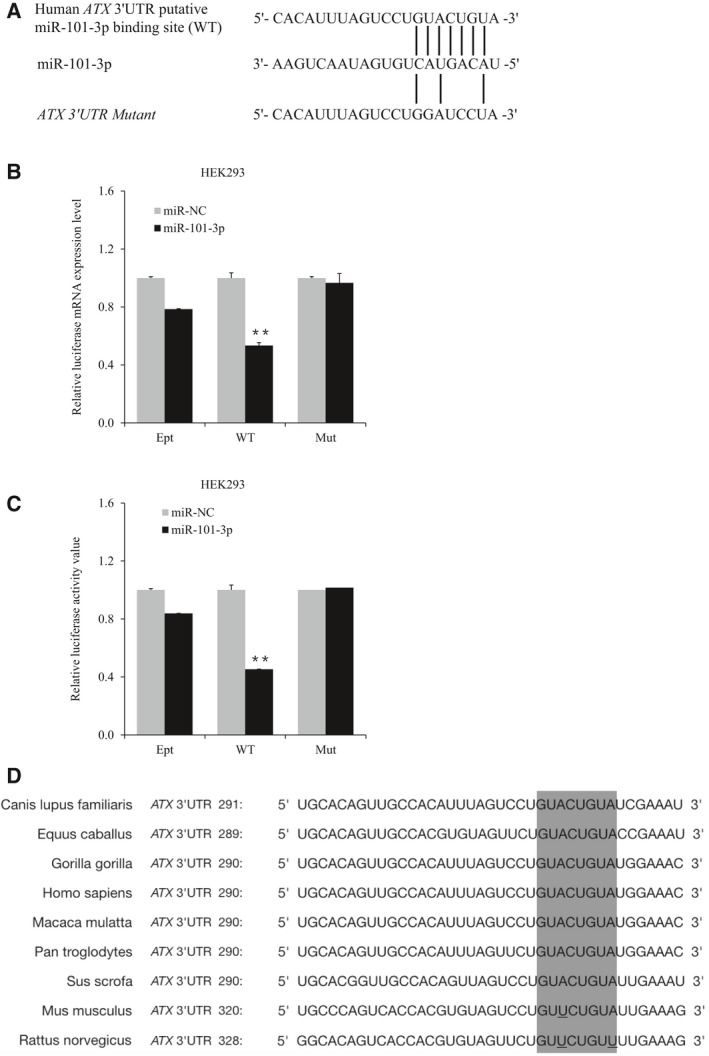
miR‐101‐3p targets the ATX mRNA 3′UTR at a conserved site. (A) A schematic diagram indicating a miR‐101‐3p binding site in the ATX mRNA 3′UTR sequence, as predicted by targetscan. Mutations were introduced to eliminate base‐pairing with the miR‐101‐3p seed sequence. (B, C) HEK293 cells were cotransfected with miR‐101‐3p mimics (miR‐101‐3p) and pRLuc (Ept), pRLuc‐ATX‐3′UTR (WT), or pRLuc‐ATX‐3′UTR‐mut (Mut). Luciferase mRNA levels (B) and luciferase activities (C) were detected 48 h after transfection. The error bars represent ± SEM. Statistical significance was determined using Student's *t*‐test. **P <* *0.01. (D) Conservation of the 8‐mer seed sequence of the miR‐101‐3p binding site (gray) within the 3′UTR of ATX mRNA was analyzed among different species. The sequences are from *Homo sapiens* (NM_001040092.2), *Pan troglodytes* (XM_009455833), *Gorilla gorilla* (XM_004047471.1), *Macaca mulatta* (XM_015145919.1), *Sus scrofa* (XM_013996524.1), *Equus caballus* (XM_014728071.1), *Canis lupus* *familiaris* (XM_014118597.1), *Mus musculus* (NM_015744.2), and *Rattus norvegicus* (NM_057104.2).

There is a predicted binding site for miR‐101‐3p in the human *ATX* 3′UTR. A mutation was made in the only predicted binding site for miR‐101‐3p in the human *ATX* 3′UTR of pRLuc‐ATX‐3′UTR to create pRLuc‐ATX‐3′UTR‐mut (Fig. 2A). In HEK293 cells, *Renilla* luciferase mRNA levels were significantly decreased when pRLuc‐ATX‐3′UTR was cotransfected with miR‐101‐3p. Nevertheless, the downregulation of *Renilla* luciferase mRNA levels was blocked by the mutation of the predicted binding site for miR‐101‐3p in the *ATX* 3′UTR (Fig. 2B). Accordingly, this mutation protected *Renilla* luciferase activity from the inhibition caused by miR‐101‐3p (Fig. 2C). Moreover, the miR‐101‐3p targeting sequence in the *ATX* 3′UTR is highly conserved among several species, while the corresponding sites in mouse and rat *ATX* 3′UTR contain one or two nonidentical nucleotides (Fig. 2D). These results suggest that miR‐101‐3p downregulates ATX expression posttranscriptionally through a conserved target sequence in the *ATX* 3′UTR, but the role of miR‐101‐3p in rodent ATX expression regulation needs to be further elucidated.

### Regulation of ATX expression by miR‐101‐3p in cancer cells

**Figure 3:**
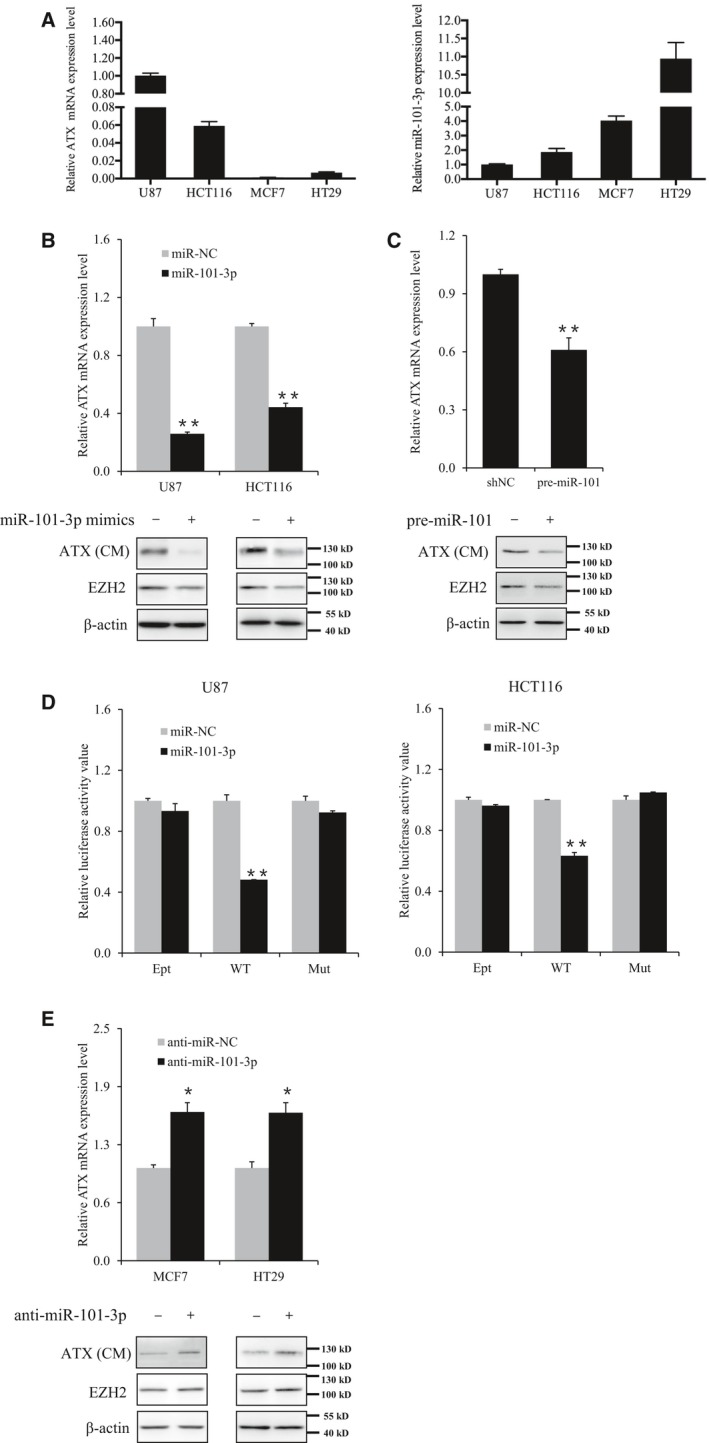
miR‐101‐3p downregulates ATX expression in cancer cells at the posttranscriptional level. (A) The expression levels of ATX mRNA and miR‐101‐3p in various cancer cell lines were detected by RT‐qPCR. (B) U87 and HCT116 cells were transfected with miR‐101‐3p mimics (miR‐101‐3p) or a negative control (miR‐NC); (C) U87 cells were transfected with the plasmid expressing pre‐miR‐101 or an empty plasmid (shNC). (D) U87 and HCT116 cells were cotransfected with the indicated luciferase reporter plasmid and miR‐101‐3p mimics or miR‐NC. Luciferase activities were detected 48 h after transfection. (E) MCF7 and HT29 cells were transfected with miR‐101‐3p inhibitor (anti‐miR‐101‐3p) or microRNA inhibitor NC (anti‐miR‐NC). Protein levels of secreted ATX in the conditional medium (CM) and EZH2 in the cells were detected by western blot, and ATX mRNA levels were detected by RT‐qPCR (B, C, E). Data are representative of three independent experiments. The error bars represent ± SEM. Statistical significance was determined using Student's *t*‐test. *P <* *0.05, **P <* *0.01.

There is an inverse correlation between ATX and miR‐101‐3p expression levels in cancer cells (Fig. 3A). In U87 and HCT116 cells with relatively high ATX expression, the expression levels of miR‐101‐3p were relatively low. Transfection of miR‐101‐3p mimics significantly reduced endogenous ATX expression in both U87 and HCT116 cells (Fig. 3B). Furthermore, to determine the effects of miR‐101‐3p in its native expressed form, U87 cells were transfected with the plasmid expressing pre‐miR‐101. It was found that the expression of ATX was downregulated in U87 cells with ectopic expression of pre‐miR‐101 (Fig. 3C). In addition, the luciferase mRNA levels in U87 and HCT116 cells harboring the pRLuc‐ATX‐3′UTR were downregulated by the transfection of miR‐101‐3p mimics. The suppression of luciferase expression by miR‐101‐3p was abolished when the seed sequence of the miR101 targeting site in *ATX* 3′UTR was mutated in the reporter plasmid (Fig. 3D), suggesting that this specific element in *ATX* 3′UTR is essential for the inhibition of ATX expression by miR‐101‐3p. In MCF7 and HT29 cells, miR‐101‐3p levels were relatively high, and ATX was expressed at a low level (Fig. 3A). ATX expression in MCF7 and HT29 cells was enhanced by suppression of miR‐101‐3p activity with a miR‐101‐3p inhibitor (Fig. 3E). The inverse correlation between ATX and miR‐101‐3p expression levels in these cancer cells is consistent with the finding that ATX is highly expressed and miR‐101‐3p is downregulated in various malignant tumors 1, 27.

### Downregulation of ATX by miR‐101‐3p suppresses cell migration, invasion, and proliferation

**Figure 4:**
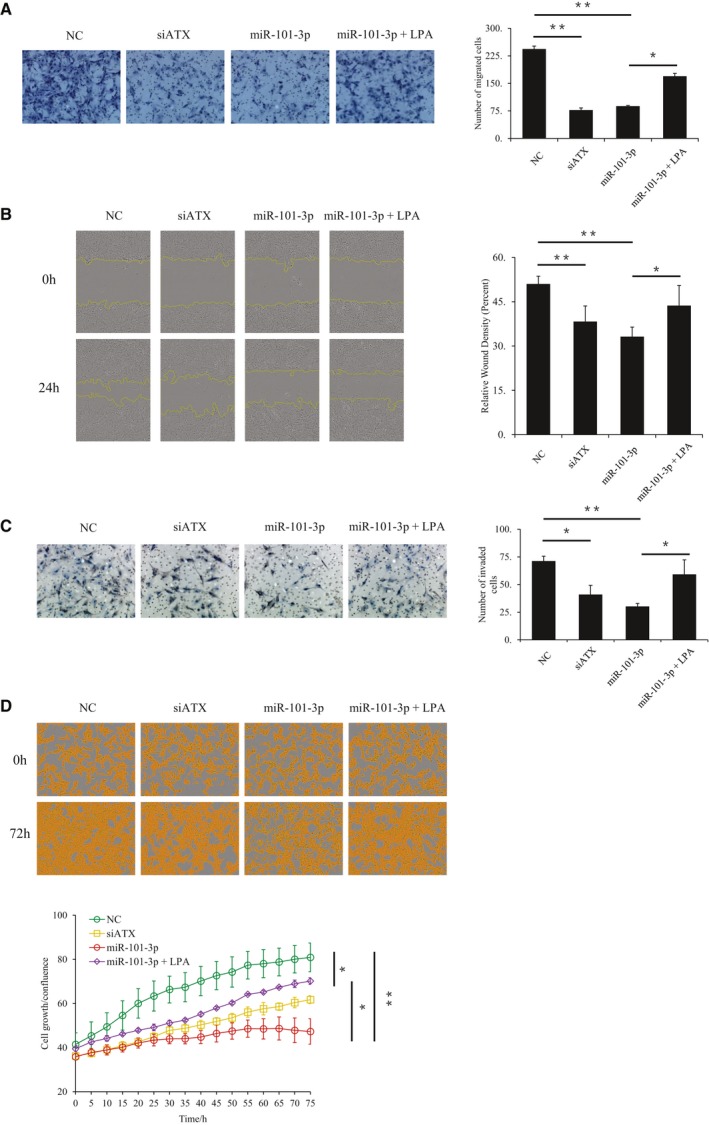
ATX downregulation contributes to the suppression of U87 cell migration, invasion, and proliferation by miR‐101‐3p. (A) Transwell migration assay. U87 cells treated with the indicated RNA duplexes in the absence or presence of LPA (2 μm) as indicated. Images of the cells on the upper chamber's lower surface were taken 24 h after the cells were seeded into the upper chamber. The relative migration was calculated by counting the cell number in the upper chamber's lower surface, and data were obtained from three randomly chosen fields. (B) Wound‐healing assay. U87 cells were treated as described in (A) and were subjected to the wound‐healing assay with the incucyte zoom longtime live cell image monitoring system. The percentage of migrated cells covering a scratch was enumerated at the indicated time points. (C) Transwell invasion assay was performed as described in (A), except that the polycarbonate membrane was coated by Matrigel before adding U87 cells to upper chamber. (D) Proliferation assay. U87 cells were treated as indicated in (A) and were subjected to the proliferation assay with the incucyte zoom longtime live cell image monitoring system. The percentage of cell growth/confluence was counted at the indicated time points. Data are representative of three independent experiments. The error bars represent ± SEM. Statistical significance was determined using Student's *t*‐test. *P <* *0.05, **P <* *0.01.

Autotaxin functions as a key enzyme to generate LPA. ATX is overexpressed in several human cancers, and the ATX–LPA axis contributes to tumorigenesis. miR‐101‐3p is a well‐known tumor suppressor that inhibits cancer cell migration, invasion, and proliferation. Since ATX is targeted by miR‐101‐3p, the effects of miR‐101‐3p‐mediated ATX downregulation on U87 cell migration, invasion, and proliferation were detected in this study. Transwell assays showed that both ATX siRNA and miR‐101‐3p treatment could significantly suppress the migration of U87 cells and that the miR‐101‐3p‐mediated suppression of migration was partially rescued by the addition of LPA (Fig. 4A). Similar results were observed in the wound‐healing assay (Fig. 4B). Matrigel invasion assays exhibited that both ATX siRNA and miR‐101‐3p treatment could suppress the invasion of U87 cells and that LPA treatment could reverse the miR‐101‐3p‐mediated inhibition of invasion to some extent (Fig. 4C). In the proliferation assay, U87 cell growth was inhibited by the miR‐101‐3p mimic or ATX siRNA treatment. The inhibition of U87 growth by miR‐101‐3p could be significantly reduced by the addition of LPA (Fig. 4D). These data suggest that the miR‐101‐3p‐mediated downregulation of ATX expression and the resulting disruption of the ATX–LPA axis contribute to the suppression of U87 cell migration, invasion, and proliferation by miR‐101‐3p.

## Discussion

MicroRNAs are short noncoding RNAs that usually function as repressors of target genes mainly by promoting mRNA degradation and/or inhibiting the translation of mRNAs at the posttranscriptional level 28. In recent years, miRNA profiling of various human cancers has identified many miRNAs that function as tumor suppressors. Genomic loss of one or both miRNA‐101 loci is observed in a variety of malignant tumors, and restoration of miR‐101 leads to the inhibition of cell proliferation, migration, and invasiveness, indicating that miR‐101 functions as a tumor suppressor 29, 30, 31. ATX is a secreted glycoprotein with LysoPLD activity that converts LPC into LPA, a bioactive lysophospholipid that regulates cell survival, migration, and differentiation through specific GPCRs. The ATX–LPA axis contributes to tumorigenesis, and ATX is regarded as a target in cancer therapy. Although ATX expression regulation in cancer cells has been studied extensively, there are few reports about ATX regulation by miRNA. Here, we demonstrate that there is a conserved miR‐101‐3p targeting element in the *ATX* 3′UTR and that ATX expression is negatively regulated by miR‐101‐3p at the posttranscriptional level. To our knowledge, this is the first report of ATX expression regulation by microRNA.

In this study, we identified ATX as a novel target for miR‐101‐3p and proved that downregulation of ATX expression contributes to the inhibition of U87 cell migration, invasion, and proliferation by miR‐101‐3p. ATX expression correlates inversely with miR‐101‐3p levels in cancer cells, which is consistent with previous reports that ATX is highly expressed and miR‐101 is downregulated in various cancers, such as neuroblastoma, breast cancer, hepatocellular carcinoma, and non‐small‐cell lung cancer. As a well‐known tumor suppressor, miR‐101 has multiple targets, including Fos, EZH2, Cox‐2, N‐myc, and Mcl‐1, that play roles in cancers 29, 30, 32, 33, 34, 35, indicating that the activity of miR‐101 is performed through various mechanisms by targeting different genes. On the other hand, the ATX–LPA axis functions through LPA receptors coupled to different G proteins (Gq, Gi, and G12/13) and their downstream molecules, such as PI3K, Ras–MAPK, Rac, and Rho. Through targeting ATX, miR‐101 could regulate multiple signaling pathways. Our findings reveal new mechanisms in ATX expression regulation and shed a novel light on the role of miR‐101 in modulating bioactive lipid signaling pathways.

## Conflict of interest

The authors declare no conflict of interest.

## Author contributions

JZ designed the experiments and analyzed the data. YW performed most experiments. LL contributed to the screening of miRNA. XZ contributed to the data analysis. JZ, XZ, and YW wrote the manuscript.
